# The Effect of Mechanical Overloading on Surface Roughness of the Coronary Arteries

**DOI:** 10.1155/2019/2784172

**Published:** 2019-01-23

**Authors:** Hanna E. Burton, Daniel M. Espino

**Affiliations:** ^1^PDR-International Centre for Design & Research, Cardiff Metropolitan University, Cardiff CF5 2YB, UK; ^2^Department of Mechanical Engineering, University of Birmingham, Birmingham B15 2TT, UK

## Abstract

**Background:**

Surface roughness can be used to identify disease within biological tissues. Quantifying surface roughness in the coronary arteries aids in developing treatments for coronary heart disease. This study investigates the effect of extreme physiological loading on surface roughness, for example, due to a rupture of an artery.

**Methods:**

The porcine left anterior descending (LAD) coronary arteries were dissected ex vivo. Mechanical overloading was applied to the arteries in the longitudinal direction to simulate extreme physiological loading. Surface roughness was calculated from three-dimensional reconstructed images. Surface roughness was measured before and after damage and after chemical processing to dehydrate tissue specimens.

**Results:**

Control specimens confirmed that dehydration alone results in an increase of surface roughness in the circumferential direction only. No variation was noted between the hydrated healthy and damaged specimens, in both the longitudinal (0.91 ± 0.26 and 1.05 ± 0.25 *μ*m) and circumferential (1.46 ± 0.38 and 1.47 ± 0.39 *μ*m) directions. After dehydration, an increase in surface roughness was noted for damaged specimens in both the longitudinal (1.28 ± 0.33 *μ*m) and circumferential (1.95 ± 0.56 *μ*m) directions.

**Conclusions:**

Mechanical overloading applied in the longitudinal direction did not significantly affect surface roughness. However, when combined with chemical processing, a significant increase in surface roughness was noted in both the circumferential and longitudinal directions. Mechanical overloading causes damage to the internal constituents of the arteries, which is significantly noticeable after dehydration of tissue.

## 1. Introduction

In the healthy left anterior descending (LAD) coronary arteries, surface roughness has recently been characterised [1]. Surface roughness can be used as a standard for the development of cardiovascular bioinspired materials used in the design of novel vascular implants for clinical treatment of vascular diseases. There is also the potential to use it to assess whether any physical or chemical changes have occurred to the surface.

Coronary artery disease is the leading cause of mortality worldwide [2]. Narrowing of the arteries such as the LAD coronary artery can result in cardiac hypoxia and impaired contractile function and increases the risk of myocardial infarction [3]. The LAD coronary artery provides a major blood supply to the myocardium [4]. Angioplasty is a procedure used to widen the blocked arteries; however, the procedure is prone to restenosis (the reoccurrence of stenosis), due to damage caused by the procedure. Inflation of the vessel can cause elastic recoil in 25-30% of patients, resulting in the narrowing of vessels at around 6 months. In more extreme cases, restenosis can occur within 24 hours of surgery due to vessel dissection or thrombus formation [5].

The mechanical behaviour of the coronary arteries can be characterised through uniaxial testing [6] which is a commonly chosen methodology for these arteries [4, 7–9]. Porcine models are typically employed because of their anatomic similarity to the human hearts [8]. The results from the uniaxial tests can be used to distinguish between the healthy and diseased arteries [10], with much interest in clinical translation via elastography [11]. There is also clinical interest in assessing the effect of mechanical overload on the arteries [9], which has implications for improved treatment outcomes of coronary artery disease.

Suitable bulk materials for coronary artery replacement are emerging [12]. There is potential for replication via emerging techniques [13], including additive manufacture of materials which are biocompatible [14]. As 30–40% of patients do not have a viable vein for replacement [15], new replacement strategies may be important. Biomaterials, though, are subject to surface degradation [16]; however, surface properties are so far mostly ignored.

The physical properties of the surfaces of the materials are usually quantified through their mean surface roughness, Ra, the arithmetic average of absolute values of sampling length [17]. Applications are typically associated with tribology and wear [18, 19], which has led to the biomedical studies of articulating tissues such as the articular cartilage [20, 21]. Recently, though, the feasibility of quantitatively measuring the surface roughness has been established for the coronary arteries [1, 22], which provides a step change from qualitative surface analysis [23]. This now opens up the potential to link mechanical overload of a coronary artery, quantitatively, with changes to its surface via Ra. Better understanding of the link between mechanical overload and changes in the surfaces has potential implications and applications clinically, including strategies for replacement.

The aim of this study was to inflict mechanical damage on the LAD coronary arteries, to mimic the initial rupture of an artery and assess changes in surface roughness. Further, chemical treatment and mechanical loading are compared to determine their effect on the surface roughness of arteries, as chemical treatment is common with the arteries [22].

## 2. Methods

### 2.1. Specimens

**Figure 1:**
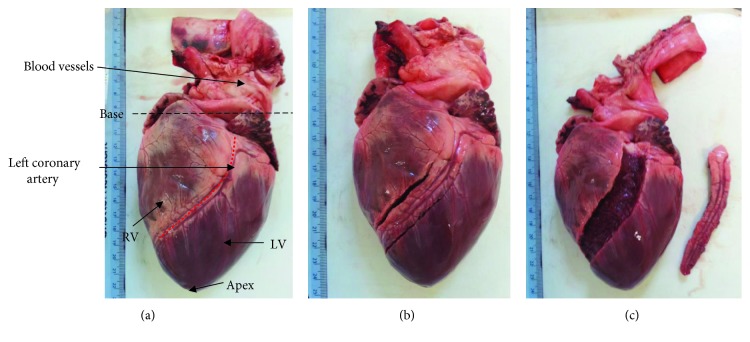
Stages of coronary artery dissection, with the (a) defrosted heart predissection, with the apex, base, blood vessels, RV (right ventricle), LV (left ventricle), and left coronary artery identified; (b) LAD coronary artery identified and dissection commenced from the most distal point visible of the LAD coronary artery; and (c) LAD coronary artery removed to the bifurcation with LCX, still attached to the myocardium.

**Figure 2:**
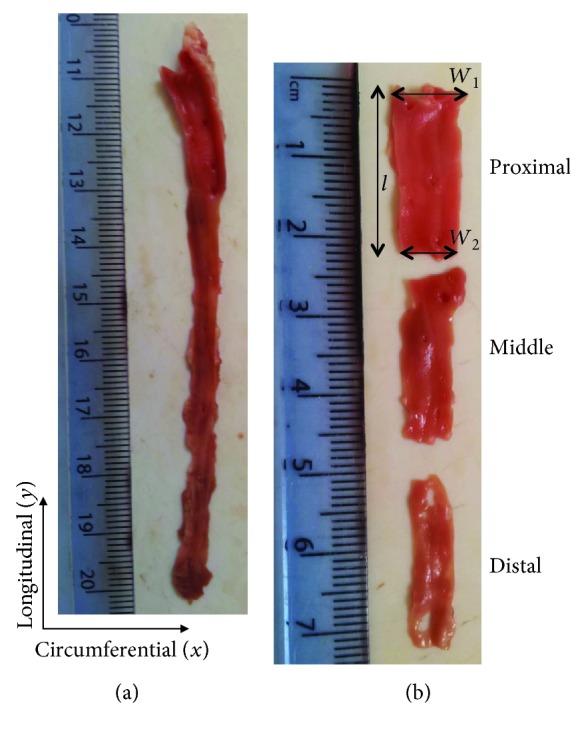
LAD coronary artery-dissected tissue with the longitudinal and circumferential axes labelled. (a) The LAD coronary artery with the myocardium removed and the LAD coronary artery opened longitudinally. (b) The LAD coronary artery specimens prepared as 20 mm sections starting at the defined top point at the bifurcation of LCX, with the proximal, middle, and distal sections identified. Length (*l*) and sample width (*W*_1_ and *W*_2_) of proximal LAD coronary artery specimens used in this study. Note: thickness (*t*) was measured perpendicular to the *x*-*y* plane, in the *z* plane.

No animals were sacrificed specifically for this study. The porcine hearts (obtained from animals approximately between 6 and 12 months old) were supplied by Fresh Tissue Supplies (Horsham, UK). Ethical approval was granted for this study by the University of Birmingham Research Support Group (ERN_15-0032). The hearts were defrosted at approximately 4°C overnight before dissection. The LAD coronary artery was identified and dissected (Figure 1) from the most distal point visible to the bifurcation of the LAD coronary artery and the left circumflex coronary artery (LCX). A longitudinal incision (along the length of the artery) was made along the LAD sample to expose its internal surface (Figure 2). Excess cardiac muscle tissue was removed from samples leaving the coronary artery tissue only. Additionally, specimens were imaged after processing, involving fixation, where for effective fixation of biological tissue the thickness of tissue samples should be less than 2-3 mm [24]. Finally, the sample was sectioned into three specimens of 20 mm each. These tissue samples were categorised as proximal, middle, and distal where in this case proximal refers to a position nearer the base of the heart and distal near to the apex of the heart, along a longitudinal axis of the LAD coronary artery (Figure 2). Dimensions of the specimens were measured along its length (*l*), width at the top and bottom (*W*_1_ and *W*_2_), and thickness (*t*) using a Vernier caliper, taking the mean of the 3 values for each dimension (Figure 2).

Tissue samples were wrapped in tissue paper soaked in Ringer's solution (Oxoid Ltd., Basingstoke, UK) and stored in heat-sealed bags at -40°C until required for microscopy. Before further testing, tissue samples were defrosted at 4°C for an hour, following protocols from previous studies of porcine heart tissue [25–28].

### 2.2. Tissue Processing

A standard protocol for fixation and dehydration of soft mammalian tissues was followed [24]. These methods for tissue processing are described in further detail elsewhere [1, 22]. Briefly, specimens were immersed in a 3% glutaraldehyde solution (Fluka Analytical, Sigma-Aldrich, St. Louis, MO, USA) with a 0.2 M sodium phosphate buffer (1 hour at pH 7.4) [29] and washed using phosphate-buffered saline (PBS) solution. Samples remained hydrated via storage in PBS solution (4°C until dehydration). Dehydration was performed by using an increased concentration of ethanol (Fisher Chemical, Fisher Scientific UK Ltd., Loughborough, UK) at 30%, 50%, 70%, 95%, and 2 × 100% [30]. Dehydration was completed using hexamethyldisilasane (HMDS; Aldrich Chemistry, St. Louis, MO, USA) [31].

### 2.3. Imaging

A noncontact, three-dimensional (3D) optical focus variation microscope (G4 Infinite Focus, Alicona UK, Kent, UK) was used to obtain three-dimensional (3D) images of the specimens at 10x magnification (10x Nikon CFI 60 TU Plan Epi Infinity Corrected Obj lens, Alicona UK, Kent, UK) and analyse their surfaces [32, 33]. Further detail and explanation of the methods used for optical imaging are provided elsewhere [1, 22]. Briefly, scans focused between the minimum and maximum heights of each sample (*z* plane) across the selected *x* and *y* sample positions (corresponding to the circumferential and longitudinal sample orientations, respectively; Figure 2). The surface analysis was performed at three distinct stages in this current study: first, when hydrated healthy; second, when hydrated damaged; and third, when damaged but following dehydration.

### 2.4. Surface Roughness

The Ra was measured for each sample using 3D reconstruction of the image via the Alicona IF-Laboratory Measurement Module (version 6.1, Alicona UK, Kent, UK). Further explanation as to this process of surface roughness measurement is provided in two preceding studies [1, 22]. Briefly, a 3D point cloud is generated following the calculation of the depth of the obtained microscopy images [34], with the entire scanned surface reconstructed. Five profile lengths (mean: 2.63 ± 0.67 mm) were measured along the *x* and *y* axes so that the Ra was assessed longitudinally and circumferentially; exclusion criteria for regions to scan were consistent with those reported elsewhere [1, 22]. Equation 1 and equation 2 [35] were used to calculate surface roughness in the circumferential direction, Ra_*C*_, and the longitudinal direction, Ra_*L*_, respectively, where *z*(*x*) is the profile height function along *x*, *z*(*y*) is the profile height function along *y*, and *l* is the sample length.

(1) $$\begin{array}{c} {Ra}_{C}=\frac{1}{l}\int_{0}^{l}\left|z\left(x\right)\right|dx, \end{array}$$

(2) $$\begin{array}{c} {Ra}_{L}=\frac{1}{l}\int_{0}^{l}\left|z\left(y\right)\right|dy. \end{array}$$

Specimens were imaged before and after damage. From the 3D reconstructed images, Ra was measured and the mean of the 5 values in the longitudinal (Ra_*L*_) and circumferential (Ra_*C*_) directions was calculated; further details on the calculation of Ra are provided elsewhere [1, 22]. Further, specimens were imaged and Ra was measured after processing involving fixation and dehydration. Middle specimens were used as a control for this study, with no damage inflicted.

### 2.5. Mechanical Testing

**Figure 3:**
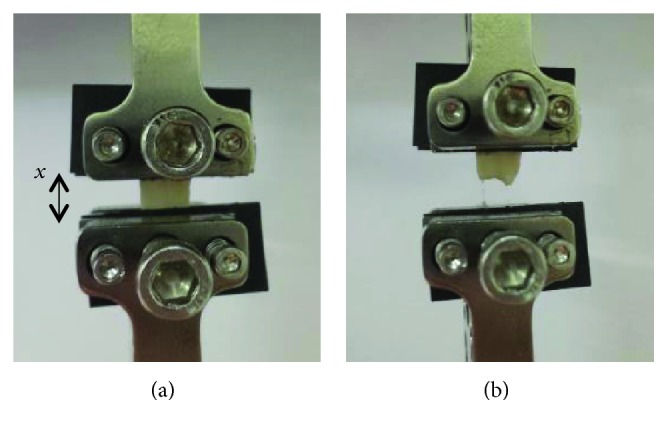
LAD coronary artery specimen under uniaxial overloading with gauge length (*x*) before failure (a) and after failure (b) due to tearing of the specimen.

Specimens were held in place for testing using grips lined with emery paper (P400 and P60) leaving an unstretched gauge length (*x*) of 4.57 ± 0.75 mm (Figure 3). The gripping method, using emery paper and a compressive force to fix the top and bottom of the specimens, is used in other studies for mechanical testing of soft, biological tissue [25, 36].

To replicate the diseased coronary arteries, damage was inflicted on specimens through uniaxial overloading of specimens using Bose ElectroForce 3200 in their longitudinal orientation. The 6 porcine hearts (*N* = 6) were dissected and the proximal and distal samples (*n* = 12) were gripped for testing. Longitudinal movement of the left coronary artery was confirmed in the range of 0.5-6.5 mm [37, 38]. Therefore, to ensure damage of the coronary artery, a displacement of 10 mm was chosen, which was identified during preliminary testing as a displacement where some specimens tore into two pieces, with a predisplacement of 4 mm on preloaded samples. A ramp rate of 1 mm/s (10% of final displacement per second) was chosen for damage of the artery, maintaining a rate within the resting physiological heart frequency range but at an elevated rate to the preconditioning. The ramp rate was applied to the specimen until a displacement of 6 mm was reached (therefore, a total specimen displacement, including predisplacement, of 10 mm). Figure 3 shows specimens before and after uniaxial overloading.

### 2.6. Statistics

The final analysis of data was performed using Minitab Statistical Software (Minitab 17.0, Minitab Inc., State College, PA, USA) on the surface roughness results of the damaged specimens. Student's *t*-test was performed to assess the significance (*p* < 0.05) under the null hypothesis of the healthy (middle specimens) and damaged (proximal and distal specimens) coronary arteries. Additionally, a paired *t*-test was used to analyse Ra of the proximal and distal specimens before and after damage to assess significance (*p* < 0.05) and to compare Ra of damaged specimens before and after processing (i.e., in their hydrated and dehydrated forms).

## 3. Results

**Figure 4:**
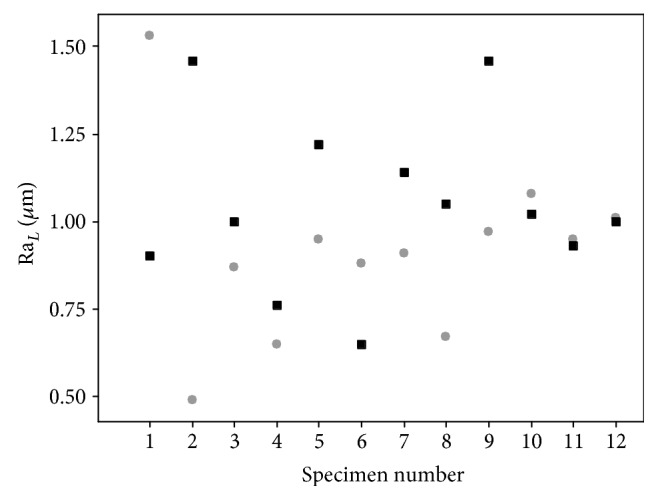
Ra_*L*_ for the individual proximal (*n* = 6) and distal (*n* = 6) specimens. Grey circles: before damage (healthy); black squares: after damage. All samples in hydrated form.

**Figure 5:**
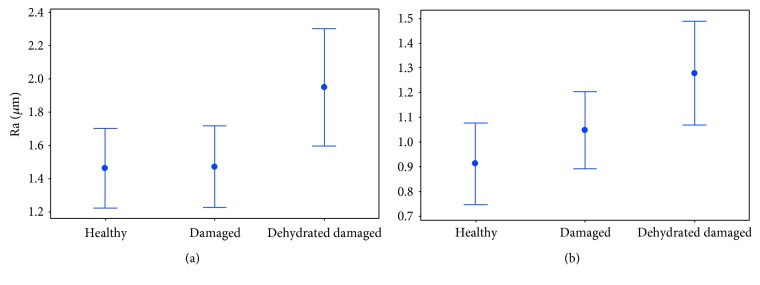
(a) Circumferential surface roughness (Ra_*C*_) and (b) longitudinal surface roughness (Ra_*L*_) of the healthy, damaged, and dehydrated damaged specimens. Error bars represent 95% confidence intervals where *n* = 12.

**Figure 6:**
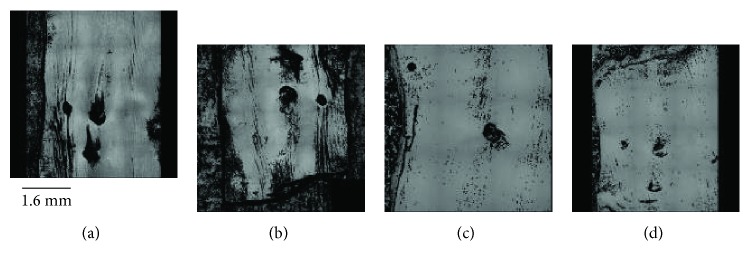
2D optical images of surfaces: (a) hydrated undamaged; (b) hydrated damaged; (c) dehydrated undamaged; (d) dehydrated damaged.

A significant difference was identified between Ra_*C*_ and Ra_*L*_ (*p* < 0.05), with surface roughness in the circumferential direction found to be greater than that in the longitudinal direction (Table 1). No significant difference (*p* > 0.05) was seen for surface roughness between the hydrated healthy control specimens (Ra_*C*_ and Ra_*L*_; 1.28 ± 0.37 and 1.00 ± 0.41 *μ*m, respectively) and the hydrated damaged proximal and distal LAD coronary arteries (Table 1). Additionally, a paired comparison of surface roughness for hydrated (proximal and distal) samples, before and after damage, revealed no significant difference (*p* > 0.05; Table 1). Individual specimen results are shown for the Ra_*L*_ in Figure 4, and although Ra_*L*_ tends to be greater after damage (Figure 5), no significant difference was noted for hydrated specimens, even when the anomalous first result is removed (Figure 4). Images of the surface in the hydrated and dehydrated forms of both the damaged and undamaged specimens are shown in Figure 6, with no visible difference between each surface.

The control undamaged specimens had a significantly greater Ra_*C*_ after processing (*p* < 0.05; 1.91 ± 0.33 *μ*m compared to 1.28 ± 0.37 *μ*m); however, Ra_*L*_ was not significantly different (*p* > 0.05; 1.07 ± 0.20 *μ*m and 1.00 ± 0.41 *μ*m), consistent with previous findings that dehydration significantly alters the surface roughness in the circumferential direction but not the longitudinal direction [22]. However, in both the circumferential and longitudinal directions of the dehydrated damaged specimens, the surface roughness was significantly greater (*p* < 0.05; Table 1) than that of the hydrated damaged specimens (Figure 5).

## 4. Discussion

This is the first study to evaluate whether there is a potential relationship between the mechanical overload of the coronary arteries, leading to failure, and their surface roughness. Although two previous studies have detailed techniques for the measurement of surface roughness, they did not assess how any mechanical overload might alter their surface roughness [1, 22]. This current study has used the recently established technique for measuring Ra of the coronary arteries [1, 22] and assessed whether it might have an application to their failure. Mechanical overload might have implications for either disease or loading due to the placement of stents.

The results of this study found a significant increase in Ra_*L*_ of the damaged LAD coronary artery when comparing chemically treated to nonchemically treated tissue, following mechanical overload. This finding differs from the results of healthy tissue studied in previous work where an increase was only seen in Ra_*C*_ [22]. In the coronary adventitia, longitudinal stiffness is a direct result of initial fibre alignment, with collagen fibres uniformly stretching in the loading direction [39]. It was hypothesised that the damage was caused by the constituents of the coronary artery, which agrees with studies by others whereby the collagen fibres are reactive to the loading of the arteries [40]. This would support the increase in surface roughness in the longitudinal direction, where collagen may have stretched and deformed under the uniaxial loading conditions. This is likely, as a significant increase was seen in Ra_*L*_ after processing, along the loading direction of the uniaxial testing. Although mechanical loading damage to the coronary arteries may not significantly alter the endothelial surface, it could affect the internal constituents of the coronary artery causing a resultant change in surface roughness. However, the constituents of soft connective tissues may alter with age [41] which may explain changes to mechanical properties of cardiovascular tissues through ageing [25]. Therefore, the specific relationship between mechanical overload and changes to surface roughness may vary during ageing; however, the generic trends identified in this current study would be expected to remain consistent across different age groups. This study demonstrates the potential of using surface roughness to assess damage and disease in the coronary arteries, with scope for future applications to assess ageing. For example, ridges were previously observed which could be altered by mechanical loading, either in orientation or profile.

The protocol for storing soft tissue by freezing used in the present study followed standard protocols used by other studies of porcine heart tissue [25, 26]. Freezing prevents the degradation of biological tissues which require storage [42]. Clark noted stiffening of vascular tissue when comparing frozen to fresh human aortic and mitral leaflets and chordae [43]. However, there was extensive overlap in results from the fresh and frozen specimens in Clark's results. Further, other studies have noted that any effects of freezing soft connective tissues are outweighed by the standard deviation of the original measurements [44]. In previous work, surface roughness of the arteries was not affected by a freeze-thaw cycle but a correction factor was necessary to correct for surface roughness when the tissue was dehydrated subsequent to glutaraldehyde-based cross-linking [22]. Cardiovascular tissues cross-linked using glutaraldehyde are known to have altered mechanical properties [45] which may explain the increase in surface roughness found in this current study.

Quantification of surface roughness properties can be combined to model disease of the coronary arteries through computational simulation. The measurements can provide a standard for bioinspired materials to adhere to, ensuring physiological similarity to native tissue. These properties are important for the development of clinical treatments through novel designs of vascular implants (e.g., stents and grafts) and tissue-engineered replacements [4]. This study also outlines a “physiological” range for surface roughness, as opposed to what may be the surface roughness of a mechanically impaired surface. These measurements, thus, should be considered during generation of bioinspired devices which are to be placed on the endothelial surface of the arteries.

It is important to consider the physiological loading conditions of biomaterials that are designed to replicate the coronary arteries. Damage can be caused by uniaxial mechanical overloading that can be noticed as an increase in surface roughness along the axis of loading. However, chemical processing, specifically dehydration, results in a significant increase in surface roughness in the circumferential direction. In other studies, changes have been noted in the surface roughness of various biological tissues due to disease [23, 46]. Therefore, a valid assumption is that disease of the coronary arteries, which can cause damage to the surface of the endothelium through the formation of atherosclerotic lesions, for example, would also result in changes to surface roughness.

Future work should investigate if mechanical damage inflicted in the circumferential direction results in an increase in Ra_*C*_, as was found for Ra_*L*_ in the longitudinal direction. Further, the combined insults to the endothelial surface from chemical processing and mechanical damage may contain further insights for assessing coronary artery changes during disease. A limitation of our current study is the use of the porcine arteries. The porcine arteries are believed to be more extendable than the human arteries, which may lead to conservative estimations for wall injury [9]. Thus, greater mechanical damage may be visible on the human arteries per given load with greater effects on surface roughness than identified in this study. However, it should be noted that most studies on the human coronary arteries use samples from older donors or patients. For example, in the above study by Van Andel et al. [9], the age of the human subjects from which the arteries were obtained ranged from 61 to 85 years old, a limitation in itself given the effects of ageing on collagen in soft connective tissues [41]. Further, a recent study has demonstrated the feasibility to objectively identify a transition point during mechanical loading of the coronary arteries [6]; the novel results presented in this current study open up the possibility to assess whether the surface roughness of the coronary arteries varies before/after such a transition.

## 5. Conclusions

In conclusion, chemical processing had a greater effect on surface roughness than mechanical damage within the specified range of testing. Mechanical testing alone did not significantly alter surface roughness. Independently, chemical processing does not affect surface roughness in the longitudinal direction (undamaged 0.91 ± 0.26 *μ*m and damaged 1.05 ± 0.25 *μ*m). When mechanical damage is inflicted in the longitudinal direction, damage is inflicted on the constituents of the coronary artery and a significant increase in Ra_*L*_ is noted after chemical processing (1.28 ± 0.33 *μ*m), but Ra_*C*_ is not affected.

## Figures and Tables

**Table 1:** Mean average result ± standard deviation of the proximal and distal samples (*N* = 6; *n* = 12) for surface roughness of the healthy and damaged LAD coronary arteries, in the hydrated and dehydrated states.

	Ra_*C*_ (*μ*m)	Ra_*L*_ (*μ*m)
Hydrated healthy	1.46 ± 0.38	0.91 ± 0.26
Hydrated damaged	1.47 ± 0.39	1.05 ± 0.25
Dehydrated damaged	1.95 ± 0.56^†^	1.28 ± 0.33^†^

† indicates the result is significantly greater than both their hydrated damaged and healthy values.

## Data Availability

The data used to support the findings of this study are available from the corresponding author upon request.
